# Supplementary material for: Fabrication of Microfluidic Chips Based on an EHD-Assisted Direct Printing Method
Source: Sensors (Basel). 2020 Mar 11;20(6):1559. doi: 10.3390/s20061559 (PMC7146459; doi:10.3390/s20061559)
Supplement: Supplementary file 1 [file sensors-20-01559-s001.zip › Suplimentary material/suplimentary material2.16.docx]

Figure S1. The relationship between the working voltage and the line width

Continuous and stable printing could be achieved between 1100 and 1800 V, but the line width was slightly different. The relationship between the line width and working voltage was shown in the figure S1.





Figure S2. The relationship between the printing speed and the line width

For the range of printing speed as 1 mm·s^-1^~40 mm·s^-1^, the relationship between the printing speed and the line width was shown in the figure S2.


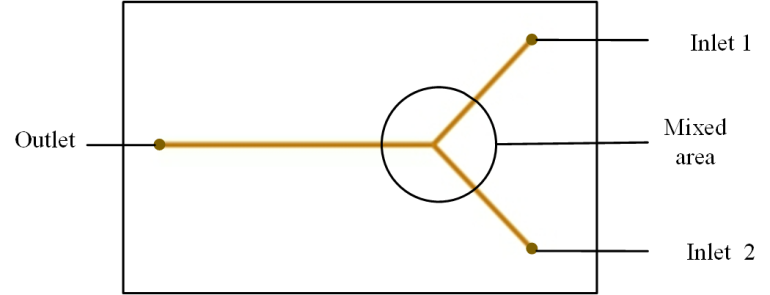


Figure S3. Schematic photo of the designed Y-type micromixer

The microfluidic chip designed for the micromixer was shown in the figuire S3 and the chip has two inlets and a mixing channel.


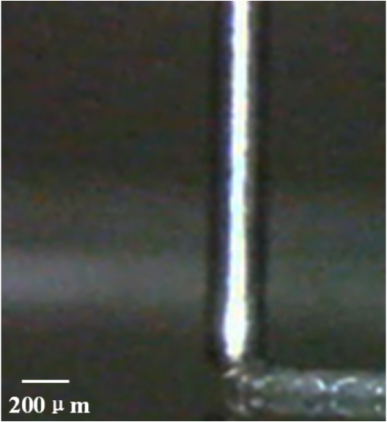


Figure S4. Photo of the nozzle in continuous cone print mode

With the increase of working voltage, the continuous paraffin wax line can be formed by utilize the middle part of the Taylor cone, and the melted paraffin wax is printed on the glass substrate as shown in the figure S4.
