# Supplementary figures and images for: Fabrication of Microfluidic Chips Based on an EHD-Assisted Direct Printing Method
Source: Sensors (Basel). 2020 Mar 11;20(6):1559. doi: 10.3390/s20061559 (PMC7146459; doi:10.3390/s20061559)

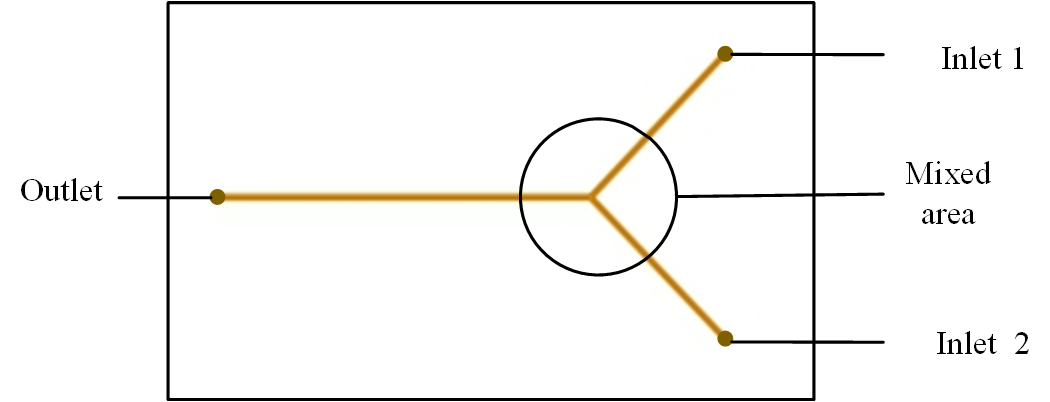

Supplement: Supplementary file 1 [file sensors-20-01559-s001.zip › Suplimentary material/Figure S3.tif]

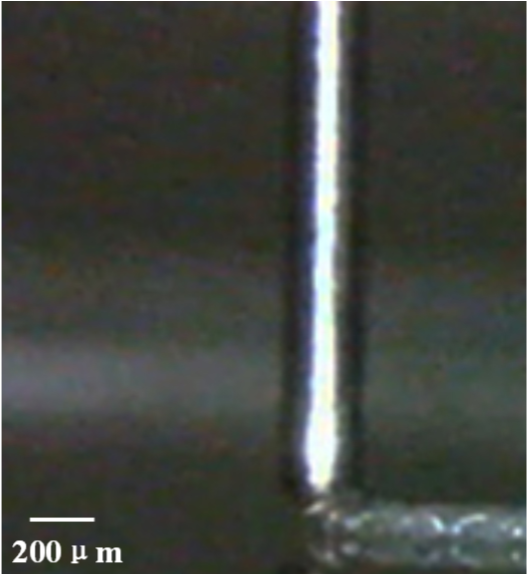

Supplement: Supplementary file 1 [file sensors-20-01559-s001.zip › Suplimentary material/Figure S4.tif]
